# Exploring AI-Assisted Query Reformulation for Older Adults Seeking Technology Support

**DOI:** 10.1093/geroni/igaf122.2179

**Published:** 2025-12-31

**Authors:** Debaleena Chattopadhyay, Hasti Sharifi, Homaira Huda Shomee, Sourav Medya

**Affiliations:** University of Illinois Chicago, Chicago, Illinois, United States; University of Illinois at Chicago, Chicago, Illinois, United States; University of Illinois at Chicago, Chicago, Illinois, United States; University of Illinois Chicago, Chicago, Illinois, United States

## Abstract

Digital applications and services have become essential for full participation in society, yet older adults often struggle to use them effectively. While quality technology support can help, many face difficulties articulating their problems due to unfamiliarity with technical terminology and age-related changes in verbal abilities. Effective communication is crucial when seeking technology support, whether from people (e.g., customer service representatives, friends, family) or systems (e.g., search engines, chatbots). This study examines these communication challenges and explores AI-driven solutions to improve older adults’ access to technology support. We conducted an online diary study with English-speaking, community-dwelling older adults (n = 27, median age = 66), to analyze asynchronous technology-related queries. Reflexive thematic analysis identified four key barriers to resolution: verbosity, incompleteness, over-specification, and under-specification. To address these issues, we designed and evaluated a few-shot prompt-chaining approach using GPT-4o. The model first elicited necessary contextual details, then paraphrased the query, and finally generated a solution. Reformulated queries significantly improved solution accuracy (69%) compared to verbatim queries (46%). Even when used solely for paraphrasing, reformulated queries yielded more accurate Google search results (69%) than verbatim ones (35%). A separate survey of digitally skilled adults (n = 48, median age = 28) found that rephrased queries were significantly more comprehensible (93%) than verbatim ones (67%). Participants also reported significantly greater confidence and ease in understanding the reformulated queries. These findings highlight the potential of AI-driven query reformulation to empower older adults in navigating digital environments more independently.

